# Intraspecific interactions in a high‐density leopard population

**DOI:** 10.1002/ece3.8227

**Published:** 2021-11-10

**Authors:** Sarah Rouse, Pouyan Behnoud, Kaveh Hobeali, Peyman Moghadas, Zolfaghar Salahshour, Hossein Eslahi, Mousa Ommatmohammadi, Ali Khani, Abolfazl Shabani, David W. Macdonald, Mohammad S. Farhadinia

**Affiliations:** ^1^ School of Geography and the Environment University of Oxford Oxford UK; ^2^ Future4Leopards Foundation Tehran Iran; ^3^ Khorasan Razavi Provincial Office of Department of the Environment Mashhad Iran; ^4^ Wildlife Conservation Research Unit University of Oxford Oxford UK; ^5^ Oxford Martin School and Department of Zoology University of Oxford Oxford UK

**Keywords:** camera trap, intraspecific interaction, multispecies occupancy model, *Panthera pardus*, paternity confusion hypothesis, temporal activity

## Abstract

Although less studied than interspecific interactions, interactions among members of the same species can influence space use and temporal activity. Using techniques commonly applied to the analysis of interspecific interactions—multispecies occupancy modeling and the analysis of temporal activity patterns—we studied intraspecific interactions within a high‐density population of Persian leopards (*Panthera pardus saxicolor*) in Tandoureh National Park, northeastern Iran. Using camera‐trap data, we investigated spatiotemporal interactions between male leopards, lone female leopards, and families (cubs/females with cubs). While we hypothesized that male and female leopards would display different temporal activity patterns, we did not predict spatial avoidance between these groups. We also predicted that leopard families would exhibit spatiotemporal avoidance from male leopards due to the risk of infanticide. Contrary to our expectations, we did not find any evidence for spatial or temporal avoidance between leopard families and adult male leopards. Male and lone female leopards exhibited positive pairwise co‐occurrence, consistent with reports of high overlap between male and female leopard home ranges. While a high level of overlap in temporal activity patterns was found between males/lone females and males/families, there was evidence for variation in the proportion of time each leopard group was active in particular periods of the diel cycle. Male leopards showed cathemeral activity, while lone females and families were more active during daylight hours. The application of these techniques to interactions within a species has improved understanding of the ecology and behavior of this endangered solitary carnivore.

## Graphical abstract

We analyzed intraspecific interactions within a high‐density population of leopards. We did not find any evidence for spatial or temporal avoidance between families and males, whereas males and lone females exhibited co‐occurrence. Male leopards showed cathemeral activity, while lone females and families apportioned more of their activity to daylight hours.

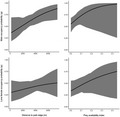

## 1 INTRODUCTION

Spatial and temporal partitioning are key mechanisms of coexistence among ecologically similar co‐occurring species (Di Bitetti et al., 2010; Santos et al., 2019; Schoener, 1974). These mechanisms are also present at an intraspecific level: Differences in feeding preferences, competition over shared resources, and avoidance of antagonistic interactions can give rise to variation in spatiotemporal activity patterns within a species (Azevedo et al., 2018; Havmøller et al., 2020).

While interactions between different species are increasingly studied using camera‐trap data (Caravaggi et al., 2017; Frey et al., 2017), the study of intraspecific interactions is an understudied but relevant application of camera‐trap data. Studies of the activity patterns of large carnivores have revealed species‐level spatiotemporal partitioning, including in the puma (*Puma concolor*) (Azevedo et al., 2018; Teichman et al., 2013), brown bear (*Ursus arctos*) (Hertel et al., 2017; Parres et al., 2020), and jaguar (*Panthera onca*) (Kanda et al., 2019).

The spatiotemporal patterns of leopard (*Panthera pardus*) behavior has been studied in the context of interactions between co‐occurring species (Carter et al., 2015; Miller et al., 2018; Steinmetz et al., 2013; Mohammadi et al., 2021), their response to human disturbance (Carter et al., 2015; Ngoprasert et al., 2007; Van Cleave et al., 2018), and prey availability (Marker & Dickman, 2005; Webb et al., 2020). The variation in spatiotemporal patterns at an intraspecific level (e.g., between age and sex groups) has not received as much attention (Havmøller et al., 2020). In leopards, intraspecific variation in space use and activity patterns can be expected due to sexual dimorphism (Farhadinia et al., 2014), differentiated energy requirements (Wilmers et al., 2017), and their polygynous mating system (Fattebert et al., 2016). For example, the larger body size of males is thought to result in males targeting larger prey compared to females (Rostro‐García et al., 2018), with the diet of female leopards consisting of a greater variety of smaller prey (Voigt et al., 2018). These contrasting prey preferences can be reflected in spatial and temporal behavior patterns (Azevedo et al., 2018).

Leopards also have a high rate of infanticide, which accounts for 40% of African leopard cub mortality (Balme & Hunter, 2013). To reduce the risk of infanticide, female leopards are known to mate with multiple males within a breeding cycle (paternity confusion) and may reduce fertility when a resident male is replaced by a new male (Balme et al., 2013). It is also expected that females with cubs reduce the risk of infanticide by avoiding adult males spatially and temporally, an adaptive strategy which is seen in other large carnivores, such as brown bears (Steyaert et al., 2013) and jaguars (Kanda et al., 2019).

Persian leopards (*Panthera pardus saxicolor*) have experienced a range contraction of up to 84% across the Middle East and the Caucasus (Jacobson et al., 2016), and now occurs mainly at low densities in montane landscapes (Ahmadi et al., 2020). Nonetheless, they reach high densities in northeastern Iran, demonstrated by 5.6 ± SD 1.0 individuals per 100 km^2^ in Tandoureh National Park (hereafter TNP; Farhadinia et al., 2019). Understanding how dense leopard populations partition their activity in space and time may shed light on the behavioral ecology of an endangered felid within an area of high human disturbance, with relevance for conservation management. For example, male jaguars are more likely to use roads and venture into agricultural land compared to females, which could put them at greater risk of human–wildlife conflict (Conde et al., 2010). As male leopards tend to move greater distances than females, similar sex‐biased threats could exist in some populations (Farhadinia et al., 2020; Van Cleave et al., 2018).

We applied multispecies occupancy modeling (Rota et al., 2016) and kernel density estimation methods (Ridout & Linkie, 2009) to investigate intraspecific spatiotemporal interactions of three groups of Persian leopard population at TNP: adult males, lone adult females, and cubs/females with cubs (hereafter, families), with these intraspecific groups considered “species” in the model. Like single‐species occupancy models, multispecies occupancy models account for imperfect detection when estimating the probability of occurrence of a species, but can also account for the effect of interactions among species on occupancy probabilities (Devarajan et al., 2020). We formulated three sets of hypotheses based on our expectations of the spatiotemporal intraspecific interactions between males/lone females and males/families and the response of these groups to a set of environmental covariates.

H1: *Male and female spatiotemporal interaction*. We hypothesized that male and lone female Persian leopards show sexual segregation in temporal activity patterns (Havmøller et al., 2020). However, we predicted that male and lone female leopards do not exhibit spatial avoidance due to evidence of high degree of intersexual overlap of home ranges in leopards (Fattebert et al., 2016; Marker & Dickman, 2005).
H2: *Male and family spatiotemporal interaction*. We predicted that leopard families differ in their spatiotemporal activity compared to lone females, exhibiting both spatial and temporal avoidance from male leopards due to the presumed high risk of infanticide (Balme & Hunter, 2013).
H3: *Response to environmental covariates*. We hypothesized that leopard family space use is positively associated with key resources—prey availability and access to water sources—while a negative response to human disturbance was expected. We did not expect such effects on male and lone female occupancy as both these groups are known to occur throughout the study area.

## 2 MATERIALS AND METHODS

### 2.1 Study site

**FIGURE 1:**
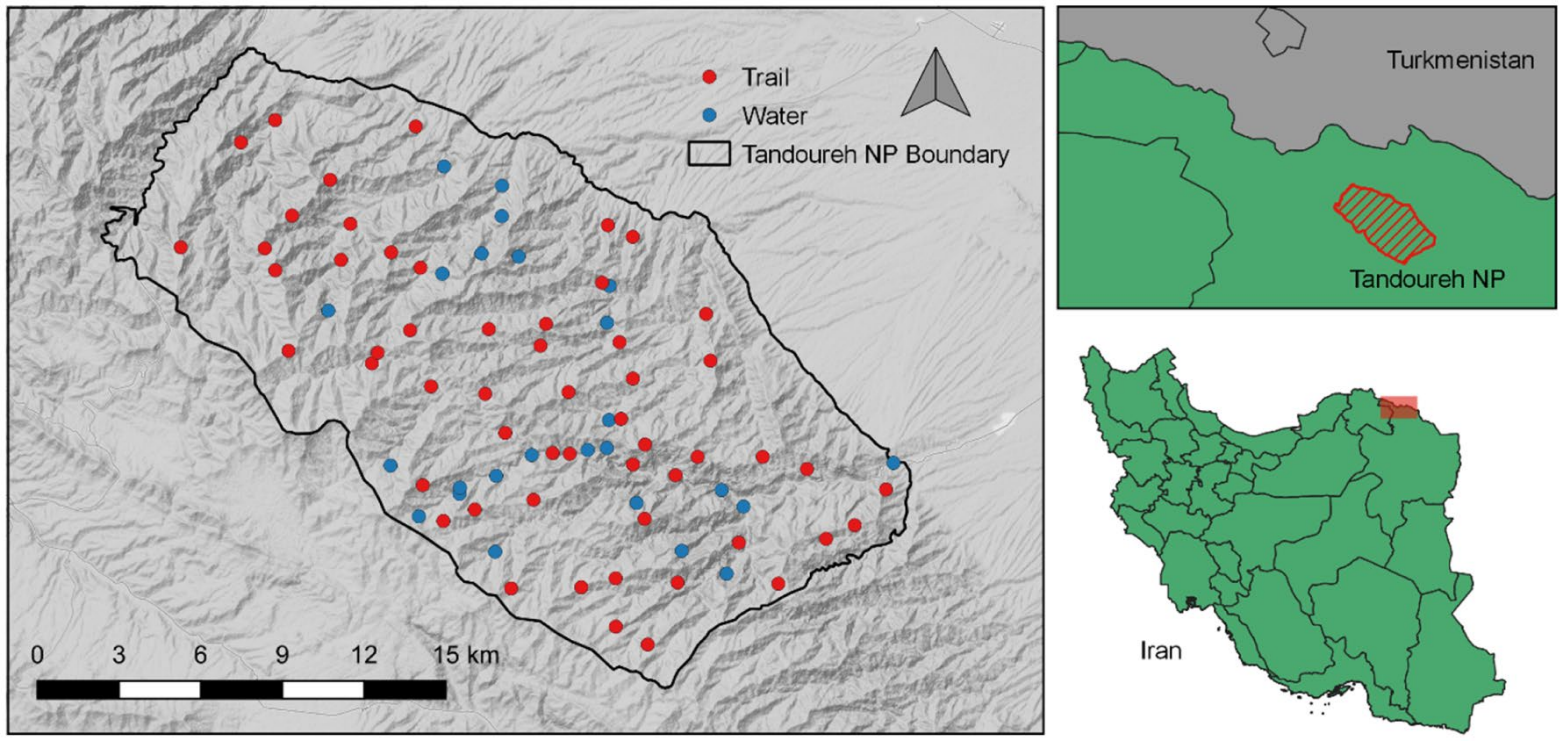
Camera‐trap placement at Tandoureh National Park, northeastern Iran. In May–July 2016, camera traps were placed at water sources (blue) or on trails (red). Maps created using QGIS version 3.14.0

**FIGURE 2:**
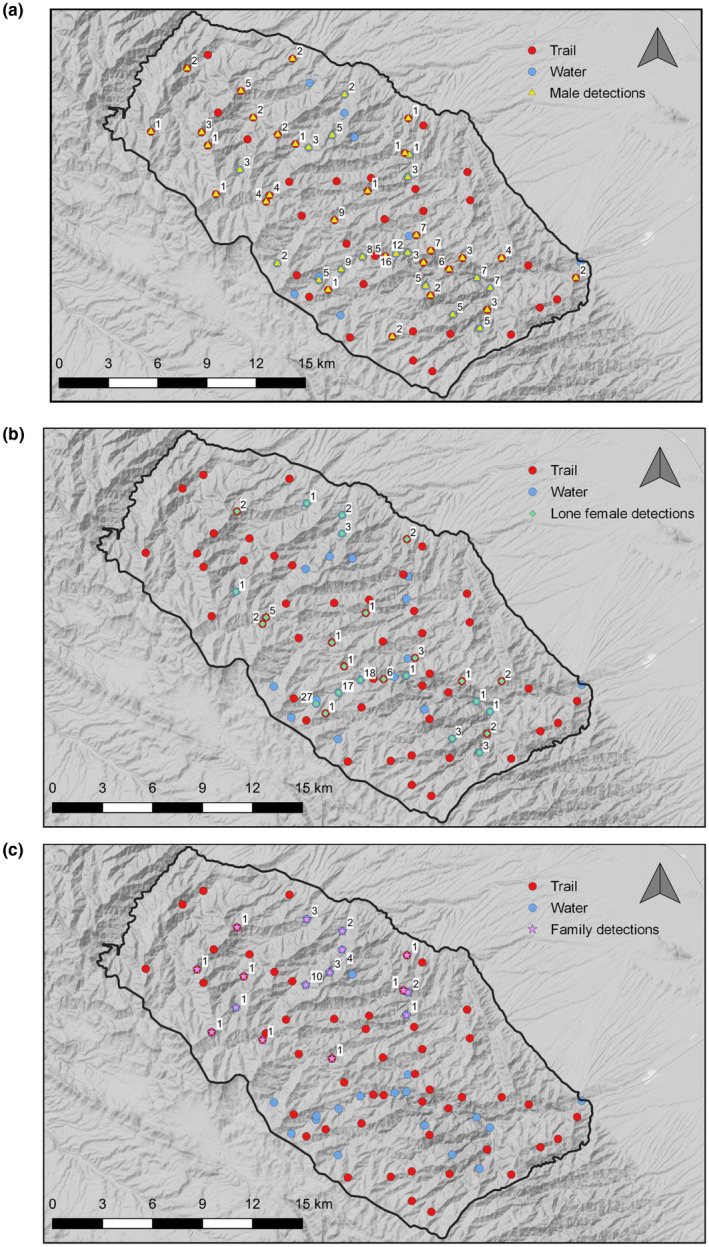
Photographic captures of the Persian leopard groups and leopard prey at Tandoureh National Park. Top panel (left to right): male leopard, lone female leopard, leopard family. Bottom panel (left to right): wild boar, bezoar goat, and urial

With an area of 355 km^2^, TNP lies approximately 20 km from the Iran–Turkmenistan border (Figure 1). The area is marked by deep valleys and cliffs, with elevation ranging from 1000 to 2600 m and experiences a temperate semi‐arid climate, with annual precipitation ranging from 250 to 300 mm (Farhadinia et al., 2018). Grazing of livestock and hunting are prohibited in areas with National Park status. The main prey of the Persian leopard available within the reserve is the wild boar (*Sus scrofa*), bezoar goat (*Capra aegagrus*), and urial (*Ovis orientalis*; Figure 2). Domestic livestock are present in the agricultural land surrounding the site (Farhadinia et al., 2018).

### 2.2 Camera‐trap sampling design

Between 31 May and 25 July 2016 during the summer and the driest period of the year, 80 Panthera IV and V Panthera^®^ IV and V (New York, NY 10018, USA) camera traps were placed along trails, predominantly along ridgelines (*n* = 55), and at water sources (*n* = 25) across TNP. An average of 2.1 cameras were placed within 3 × 3 km park‐wide grids, at a mean spacing of 1220 m (±SE 63) between each camera‐trap station. Camera traps were placed approximately 40 cm off the ground on natural features, on either trees or rock piles. Trails and water sources were selected due to their accessibility and to maximize leopard detections: Leopards at TNP are thought to travel along ridgelines and valley bottoms rather than using cliff faces, and water‐based sampling can maximize detection of more elusive groups including females and cubs (Farhadinia et al., 2019).

### 2.3 Data preparation

Individuals were sexed and assigned to categories of male/lone female/family and if possible, identified as individuals based on distinctive rosette patterns on their pelage (Figure 2). Leopards were sexed based on sex‐specific characteristics, for example, genitalia or presence of cubs (<1 year old). Female leopards, which were known to have cubs but were photographed alone, were classed as belonging to the “family” category due to the possibility that mothers were being accompanied by cubs, which had not been detected by the camera trap. Due to the immobility of leopard cubs in the first few months of life, it is possible that leopard mothers with very young cubs were not detected in our survey. Multiple detections at the same camera‐trap station <30 min apart were removed from the dataset to ensure independence of detections (Linkie & Ridout, 2011).

### 2.4 Statistical methods

All spatial and temporal analyses were carried out in R version 4.0.2 (R Core Team, 2020).

#### 2.4.1 Multispecies occupancy models

To simultaneously analyze the occupancy probability of leopard groups in response to both environmental variables and the presence or absence of conspecifics, we fitted two sets of multispecies occupancy models for (a) male leopards and lone females and (b) male leopards and families.

We used the multispecies occupancy model for two or more interacting species by Rota et al. (2016). This model is usually applied to investigate patterns of spatial co‐occurrence between species, but also has potential to be used for the analysis of intraspecific co‐occurrence patterns of different groups within the same species. Models were fit using the “occuMulti” function in the R package *unmarked* (Fiske & Chandler, 2011), which implements the Rota et al. (2016) model using a maximum likelihood estimation approach built into the package. Building upon the single‐species, single‐season occupancy model (MacKenzie et al., 2002), the Rota et al. (2016) multispecies occupancy model accounts for imperfect detection while allowing for the simultaneous analysis of the effect of environmental covariates and interacting species on occupancy probability.

One of the main advances of this model upon previous multispecies models is that there is no need to assume that species within the model are either dominant or subordinate, which was a potential limitation of previous iterations of multispecies occupancy models (Rota et al., 2016). In each model, which included two “species,” we modeled the possible occupancy states for each pair of leopard groups with 0 indicating nondetection and 1 indicating detection. The model produces both marginal (without interactions) and conditional (including interactions) occupancy probabilities (Table A1: Appendix S1).

Before model fitting, we constructed detection histories for each group. Detection histories were entered into an M × J design matrix consisting of 1s and 0s indicating detection and nondetection, where M = number of camera‐trap stations (*n* = 79) and J = number of sampling occasions (*n* = 56 days). A leopard was considered detected during the sampling period if it was captured at least once during a full 24‐h period.

As our main goal was to determine the potential effect of intraspecific leopard interactions on leopard occupancy, candidate models were kept simple. We built two small sets of candidate models for each leopard pair (males/females and males/families), which included covariates based on a priori biological hypotheses which we expected to affect leopard occupancy and detection probability (Table A2: Appendix S1). Five covariates were chosen to explain potential variation in leopard occupancy. Latitude and longitude were included as geographical control covariates (Miller et al., 2018; Rota et al., 2016), and three environmental covariates were selected: prey availability (Prey), distance to park edge (DistEdge) as proxy for human disturbance, and whether the leopard was captured at a trail or water source (Water/Trail). Prey availability was calculated by counting the number of photos at each camera‐trap station of the main prey items for leopards in the area (wild boar, bezoar goat, urial). Following our approach for leopard detections, we considered prey detections independent if photographs were taken >30 min apart (Linkie & Ridout, 2011). These counts were then standardized by the number of days the camera was active to produce an index of prey availability, which was taken as a biologically representative proxy of prey abundance. Two covariates were used to model detection probability for all three leopard groups: Whether the camera trap was placed at a trail or water source (Water/Trail) and the number of days the camera was active over the study period (DaysActive).

We adopted a two‐step approach to building our occupancy models. Prior to building candidate models of occupancy, we determined the best fitting model for detection probabilities for each leopard group while assuming constant occupancy. Candidate models were ranked using Akaike information criterion (AIC), with models with a difference in AIC (ΔAIC) < 2 from the model with the lowest AIC score considered to carry substantial support (Burnham & Anderson, 2002; MacKenzie & Bailey, 2004). The potential associations between the covariates and occupancy were assessed using 95% confidence intervals, with intervals which overlapped with 0 taken to indicate that the covariate did not affect occupancy.

The best fitting detection model for all leopard groups was one which included both our detection covariates (Water/Trail and DaysActive); therefore, these detection covariates were carried over in all of the subsequent candidate occupancy models. For the male/lone female multispecies occupancy model, eight candidate models were run which included null models with and without an interaction term and global models including all covariates. Due to the smaller sample size of families and risk of overparametrization, candidate models for the male/family multispecies occupancy model were built by starting with a null model and iteratively adding covariates. Models with 2+ covariates either failed to converge or produced large coefficient estimates with wide confidence intervals, which overlapped with zero; therefore, global models were not included in model ranking.

Five candidate models were then tested for the male/family multispecies occupancy model. All interactions were set at the intercept, meaning we assumed male/lone female and male/family interaction was constant along environmental covariates. This was due to a lack of a priori hypotheses for predicting variation in interaction strength along our environmental covariates. Model‐averaged estimates of occupancy probability from top‐ranking occupancy models (ΔAIC < 2.00) were estimated using the “mogavgPred” function in the *AICcmodavg* package (Mazerolle, 2020) to account for uncertainty in occupancy probability estimates among these top models.

Finally, as spatial avoidance patterns of leopard families might be expected to differ in response to leopard fathers compared to nonfathers, we calculated the average number of unique males detected at stations where families had been detected and compared this to stations where families were absent. If more than one male was visiting family stations, this could indicate that male leopards other than fathers were frequenting sites also occupied by families.

#### 2.4.2 Temporal activity patterns

Detection times were first converted to sun time following the recommendations of Nouvellet et al. (2012). Clock times often do not reflect the actual timing of astronomical events such as sunset and sunrise; therefore, sun time is recommended for the analysis of animal activity patterns to reduce noise. This conversion was carried out using the “sunTime” function in the R package *overlap*, which converts clock time to sun time using sunrise and sunset data from the National Oceanic & Atmospheric Administration.

We then divided the 24‐h day into diurnal, nocturnal, and crepuscular periods based on the average sunrise and sunset time at TNP. To do this, we calculated mean sunrise and sunset times over the study period, using the “daylength” function in the R package *insol* (Corripio, 2014). The mean timing of sunrise was 05:10, while mean sunset time was 19:40. The crepuscular periods were defined as 1.5 h before and after sunrise and sunset (03:41–06:39 and 18:11–21:09), based on the approximate length of astronomical twilight, nautical twilight, and civil twilight (timings from www.timeanddate.com) to ensure all crepuscular activity was captured. Therefore, 1.5 h after sunrise to 1.5 h before sunset was considered the diurnal period (06:40–18:10), while nocturnal hours were defined as 1.5 h after sunset to 1.5 h before sunrise (21:10–03:40). A Chi‐squared test was performed in R to determine whether the observed proportions of detections for males, lone females, and families across the three time periods (diurnal, nocturnal, and crepuscular) differed significantly from the expected proportions for each time period.

Daily activity patterns of leopards could then be categorized as either diurnal (<10% detections at night), mostly diurnal (10%–29% detections at night), nocturnal (≥90% detections at night), mostly nocturnal (70%–89% detections at night), or crepuscular (50% detections during dawn or dusk), with any falling outside of these categories classed as cathemeral (Azevedo et al., 2018; Gómez et al., 2005).

To compare intraspecific overlap in daily activity patterns, we used the widely applied nonparametric kernel density estimation method for circular data developed by Ridout and Linkie (2009). Analyses were carried out in the R package *overlap* (Meredith & Ridout, 2020). After converting time to radians, a probability density curve was produced for each leopard group. The degree of temporal overlap between leopard groups was then estimated with the coefficient of overlapping, ∆, where a value of 0 represents no overlap and 1 represents complete overlap. Meredith and Ridout (2020) suggest three variants of the ∆ estimator (∆_1_, ∆_4_, and ∆_5_) and recommend using ∆_1_ when the number of photographic detections is <50, and ∆_4_ when detections >75. As the number of detections of families was below 50 (*n* = 34), the ∆_1_ estimator was used for male/family overlap estimation and ∆_4_ for male/lone female overlap, for which we had over 100 detections for each sex. Following recommendations in Meredith and Ridout (2020), a smoothing parameter of 1 was used for ∆_4_ and a smoothing parameter of 0.8 was applied to overlap estimates using ∆_1_. We calculated 95% confidence intervals for each overlap estimate from 10,000 smoothed bootstrap samples.

As the coefficient of overlap is a descriptive statistic, Watson's two‐sample *U* ^2^ test for circular data was performed using the “circular” package in R (Lund et al., 2017) to calculate significance estimates between density curves of the leopard groups.

## 3 RESULTS

**FIGURE 3:**
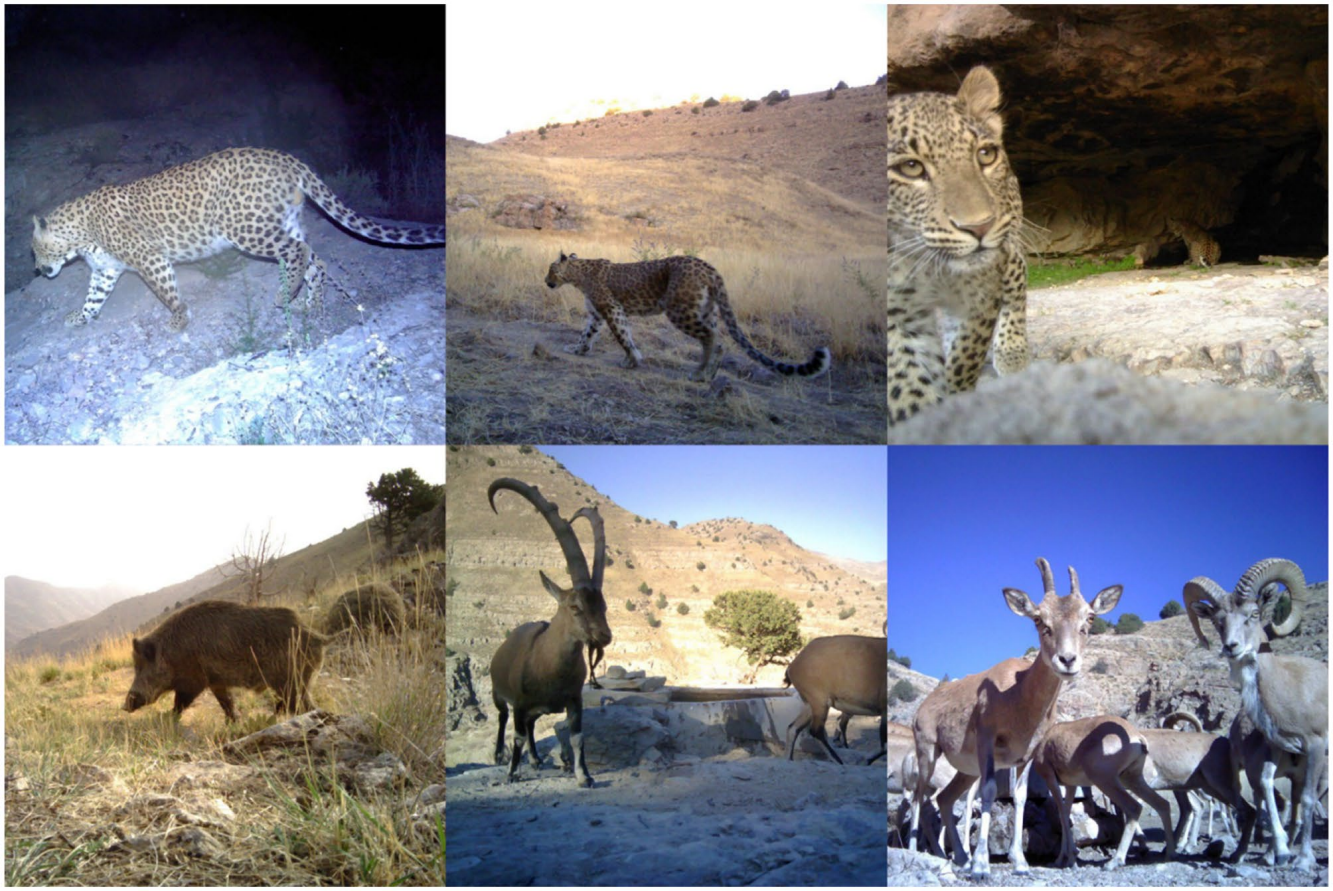
(a–c) Detections of each leopard group at camera‐strap stations in Tandoureh National Park; males (a), lone females (b), and families (c)

Over the sampling period spanning 31 May to 25 July 2016, 343 independent detections of leopards were captured at 49 out of 80 camera‐trap stations at TNP. Of these 343 detections, 183 were identified as male, 107 were females and 34 were classed as families (meaning either detections of cubs, or females known to have cubs; Figure 3a–c). 19 unique males, 16 lone females, and 5 family groups were identified during the sampling period. 19 independent detections belonging to individuals where sex could not be determined were excluded from analysis, leaving 324 independent detections. Of the 80 camera traps established for the survey, 1 trap was removed from data analysis as it was inactive throughout the study period.

### 3.1 Multispecies occupancy models

**FIGURE 4:**
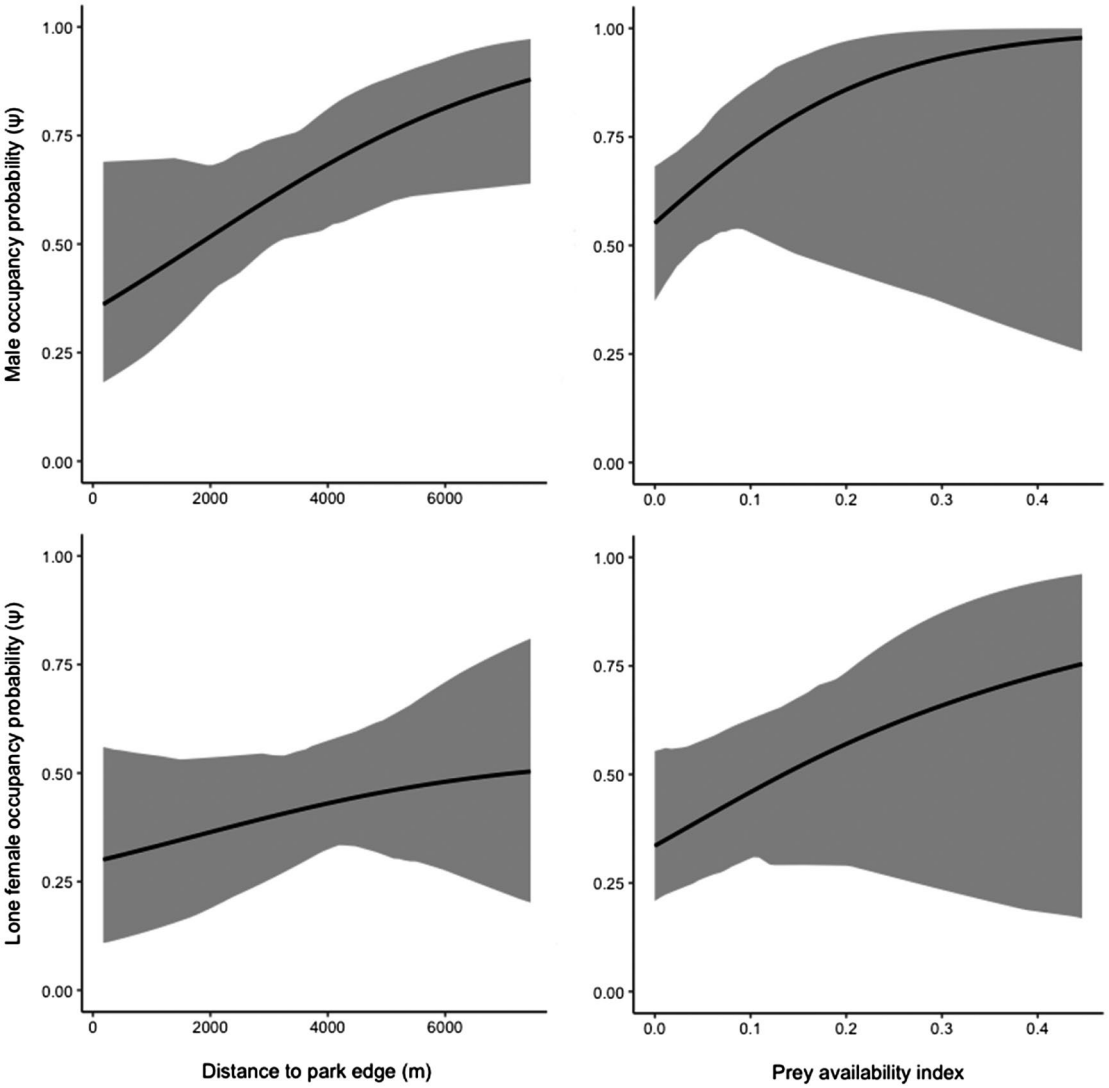
Plotted model‐averaged marginal occupancy probabilities (occupancy without taking into account species interactions) of male leopards and lone female leopards as a function of distance to the edge of Tandoureh National Park (column 1) and prey availability (column 2), with values for other environmental covariates held constant. Gray ribbons indicate 95% confidence intervals. Due to the very large confidence intervals for family occupancy, marginal occupancy plots for leopard families were excluded

Male leopards had the highest marginal occupancy probabilities (occupancy without interactions) in the study area (Ψ = 0.67, CI: 0.42–0.92), followed by lone female leopards (Ψ = 0.42, CI 0.09–0.82) and leopard families (Ψ = 0.41, CI: 0.09–0.88; Figure 4).

#### 3.1.1 Male and lone female co‐occurrence

The top three ranking models (ΔAIC < 2.00) all included a pairwise interaction term (Table 1). Model‐averaged conditional occupancy probabilities showed occupancy probability was highest when both males and lone females were present: Ψ_11_ (male present/lone female present) = 0.41, CI: 0.15–0.68; Ψ_10_ (male present/lone female absent) = 0.29, CI: 0.09–0.48; Ψ_01_ (male absent/lone female present) = 0.043, CI: 0.06–0.14. Male and lone female occupancy showed positive pairwise covariance, which was consistent with our hypothesis (H1; M1: β_male/lone female_ = 2.09, CI: 0.35–3.82).

**TABLE 1:** Model structure of candidate models run for analysis of male/lone female occupancy analysis

	Model structure	K	AIC	ΔAIC	AICwt
M1	ψ_male_(DistEdge) ψ_lone female_(DistEdge) ψ_male/lone female_(.) p_male_(DaysActive, Water/Trail) p_lone female_(DaysActive, Water/Water/Trail)	11	1966.27	0.00	0.34
M2	ψ_male_(.) ψ_lone female_ (.) ψ_male/lone female_(.) p_male_(DaysActive, Water/Trail) p_lone female_(DaysActive, Water/Trail)	9	1966.68	0.41	0.27
M3	ψ_male_(Prey) ψ_lone female_ (Prey) ψ_male/lone female_(.) p_male_(DaysActive, Water/Trail) p_lone female_(DaysActive, Water/Trail)	11	1966.75	0.48	0.26
M4	ψ_male_(Water/Trail) ψ_lone female_(Water/Trail) ψ_male/lone female_(.) p_male_(DaysActive, Water/Trail) p_lone female_(DaysActive, Water/Trail)	11	1968.90	2.22	0.12
M5	ψ_male_(.) ψ_female_(.) p_male_(DaysActive, Water/Trail) p_lone female_(DaysActive, Water/Trail)	8	1972.47	5.78	0.02
M6	ψ_male_(Lat + Long) ψ_female_(Lat + Long) ψ_male/lone female_(.) p_male_(DaysActive, Water/Trail) p_lone female_(DaysActive, Water/Trail)	13	1972.59	5.91	0.019
M7	ψ_male_(Global), ψ_female_(Global), ψ_male/lone female_(.) p_male_(DaysActive, Water/Trail) p_lone female_(DaysActive, Water/Trail)	19	1978.97	12.29	0.0007
M8	ψ_male_(Global), ψ_female_(Global) p_male_(DaysActive, Water/Trail) p_lone female_(DaysActive, Water/Trail)	18	1983.86	17.18	0.000067

Note

Models with ΔAIC < 2 were considered to have substantial support. ψ = occupancy probability, *p* = detection probability, *k* = number of parameters, AIC = Akaike Information Criterion, ΔAIC = difference in AIC value compared to the top‐scoring model, ΔAICwt = the probability the model is the top model, relative to all other candidate models.

While top‐scoring models M1 and M3 included environmental covariates (DistEdge and Prey), the confidence intervals of these effects overlapped with 0, which indicated a lack of effect of these covariates on male and lone female occupancy. We did not find any evidence that occupancy of males or lone females was affected by whether the site was a water source or trail. Therefore, there was no evidence that the inclusion of environmental covariates had an effect on the occupancy of these groups, potentially due to high occupancy of male and lone female leopards throughout the study site.

#### 3.1.2 Male and family co‐occurrence

Three of the five candidate models tested for males and families had ΔAIC <2.00, meaning they held equal support: M9, a null model assuming constant occupancy for males/families with no pairwise interaction; M10, a model which assumed family occupancy was a function of the Water/Trail covariate with an male/family interaction term; and M11, a null model which included a constant pairwise interaction term between males and families (Table 2). There is therefore considerable uncertainty in making inferences about male leopard and family interaction based on these model outputs, as models with and without an interaction term held equal support. To add to this uncertainty, top‐ranking model M10 reported negative pairwise co‐occurrence (M10: β_male/family_ = −0.51, CI: −2.14 to 1.11), while the interaction coefficient produced by M11 indicated positive pairwise co‐occurrence (M11: β_male/family_ = 0.99, CI: 0.86–2.85).

**TABLE 2:** Model structure of candidate models run for analysis of male/family occupancy analysis

	Model structure	K	AIC	ΔAIC	AICwt
M9	ψ_male_(.), ψ_family_(.) p_male_(DaysActive, Water/Trail) p_family_(DaysActive, Water/Trail)	8	1569.94	0.00	0.36
M10	ψ_male_(.), ψ_family_(Water/Trail), ψ_male/family_(.) p_male_(DaysActive, Water/Trail) p_family_(DaysActive, Water/Trail)	10	1570.52	0.58	0.27
M11	ψ_male_(.), ψ_family_(.), ψ_male/family_(.) p_male_(DaysActive, Water/Trail) p_family_(DaysActive, Water/Trail)	9	1570.82	0.88	0.23
M12	ψ_male_(.), ψ_family_(Prey), ψ_male/lone female_(.) p_male_(DaysActive, Water/Trail) p_family_(DaysActive, Water/Trail)	10	1572.24	2.31	0.11
M13	ψ_male_(.), ψ_family_(DistEdge), ψ_male/family_(.) p_male_(DaysActive, Water/Trail) p_family_(DaysActive, Water/Trail)	10	1575.61	5.67	0.02

Note

Models with ΔAIC < 2 were considered to have substantial support.

Model‐averaged occupancy probabilities indicated family occupancy was not negatively impacted by the presence of male leopards: Ψ_11_ (male present/family present) = 0.34, CI: 0.042–0.63; Ψ_10_ (male present/family absent) = 0.34, CI: 0.0075–0.68; Ψ_01_ (male absent/family present) = 0.15, CI: −0.0445 to 0.34. Based on these models, we did not find support for our hypothesis (H2) that leopard families exhibited spatial avoidance; although M10 reported a negative interaction, this coefficient had very wide confidence intervals overlapping 0. In terms of environmental covariate predictions (H3), these results did not support our expectation that leopard family occupancy would be negatively associated with increased proximity to the edge of the park. Furthermore, the inclusion of covariates which were representative of resources which we predicted would be important for families (Prey and Water/Trail) did not improve model fit.

The average number of unique males detected at stations where families were also detected throughout the study period was 1.31 (±SD 0.98) males, compared to 1.72 (±SD 0.80) unique males detected at nonfamily stations. In addition, out of the 16 camera traps where families were detected, 9 were visited by ≥2 unique males.

### 3.2 Temporal activity patterns

During the study period, 42.1% of leopard detections occurred during nocturnal hours (20:40–04.10), 33.6% during diurnal hours (06:10–18:40), and 26.2% during crepuscular hours (04:11–06:09 and 18:40–20:39). Following circadian activity categories defined by Gómez et al. (2005) and Azevedo et al. (2018), this species‐level distribution of detections corresponded to a cathemeral activity pattern, where activity is distributed approximately evenly between the light and dark periods of the day. However, there was evidence of different proportions of detections for each leopard group within each time period (χ^2^ = 14.482, *df* = 4, *p* = .0059; Table 3). At the intraspecific level, males tended to be “cathemeral,” whereas lone females and families were “mostly diurnal.” Families had the highest proportion of daily activity apportioned to the crepuscular time period (41%).

**TABLE 3:** Number of detection records for males, lone females, and families which fell within time periods defined as nocturnal, diurnal, or crepuscular (% of records for each leopard group)

	Nocturnal (21:10–03:40)	Diurnal (06:40–18:10)	Crepuscular (03:41–06:39, 8:11–21:09)
Males (*n* = 183)	80 (44%)	42 (23%)	61 (33%)
Lone females (*n* = 107)	29 (27%)	43 (40%)	35 (33%)
Families (*n* = 34)	8 (24%)	12 (35%)	14 (41%)

**FIGURE 5:**
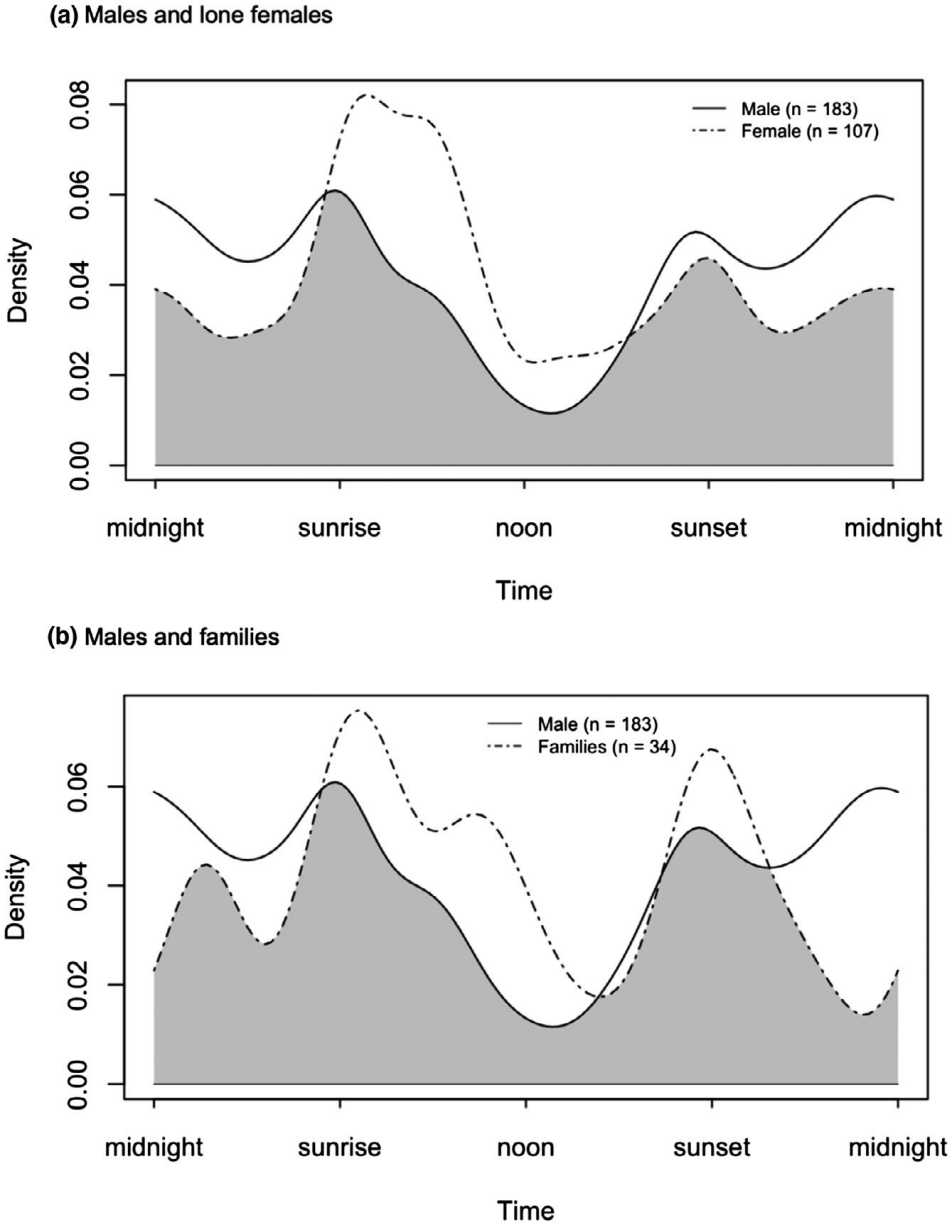
Temporal overlap plot of (a) male leopards (solid line) and lone female leopards (dashed line; ∆4 = 0.80), and (b) male leopards (solid line) and leopard families (dashed line; ∆1 = 0.79). Gray areas underneath density curves represent the overlap coefficient, ∆

Overall, all leopard groups displayed two activity peaks to varying extents at sunrise and sunset, with leopard families showing a third peak in activity prior to noon. The coefficient of overlapping indicated a high degree of overlap (∆_4_ = 0.80 [CI = 0.71–0.89]) between males and lone females (Figure 5), with evidence of different activity curves (*U* ^2^ = 0.338, *p* < .05). A similarly high degree of overlap was also present for males and families (∆_1_ = 0.79 [CI = 0.66–0.91]; Figure 4); however, there was no evidence for differences in the activity curves between this pair (*U* ^2^ = 0.178, *p* > .05).

## 4 DISCUSSION

### 4.1 Spatial co‐occurrence patterns

This study provides a novel application of the Rota et al. (2016) multispecies occupancy model to an analysis of spatial co‐occurrence at an intraspecific level. Our top models of male and lone female occupancy included a pairwise interaction, suggesting that accounting for intraspecific interactions in occupancy modeling studies can improve understandings of the drivers of space use.

In accordance with our expectations, males and lone females exhibited strong positive pairwise covariance (M1: β_male/lone female_ = 2.09, CI: 0.35–3.82), which is consistent with studies reporting a high degree of intersexual home range overlap in leopards (Fattebert et al., 2016; Marker & Dickman, 2005). Intraspecific interaction was a strong associate of leopard space use compared to other covariates such as prey availability, human disturbance, and camera placement (water source/trail). However, these associations can arise due to the similar habitat preferences shared by individuals of the same species as opposed to the presence of each group influencing the occupancy of the other.

Spatial and temporal analyses of leopard cubs are often not possible due to the difficulties in obtaining an adequate number of detections of cubs for analysis (du Preez et al., 2014). Due to the high number of detections of families from our dataset relative to other studies (e.g., 2500 unbaited camera trapping occasions yielded zero leopard cub detections in Zimbabwe; du Preez et al., 2014), we were able to offer insights into the space use and temporal activity of leopard families. Contrary to our expectations, within this Persian leopard population we did not find any evidence for spatiotemporal avoidance between male leopards and leopard families, despite the apparent high risk of infanticide presented by roaming males (Balme & Hunter, 2013). This can be explained in a number of ways.

First, the physical features of the study area may facilitate spatial intraspecies coexistence. The rugged terrain of TNP, characterized by steep cliffs and valleys, could contribute to the high spatial overlap among conspecifics (Farhadinia, Heit, et al., 2020). The physical heterogeneity of the landscape could allow leopard families to live in close proximity to males due to the presence of features, which provide concealment compared to flat landscapes such as savannah. Furthermore, the relatively small size of the national park (355 km^2^) coupled with a high‐density leopard population (5.6 ± SD 1.0 individuals/100 km^2^; Farhadinia et al., 2019) may limit the extent to which leopard families can avoid male leopards. For example, Penteriani et al. (2020) found that in a confined brown bear population with limited habitat options, females with cubs did not avoid males at large spatial scales despite the threat of infanticide. Therefore, spatial avoidance may have occurred at finer spatial scales that our study was unable to capture (Broekhuis et al., 2014), with the coarse coverage of the study area potentially insufficient to reveal finer‐scale habitat preferences.

Alternatively, male leopards, which overlap in space and time with families, may have sired the cubs, therefore would not present a threat to cubs. However, the average number of unique males frequenting stations where families were detected compared to stations where families were absent were similar—notwithstanding the possibility of litters with multiple paternity (i.e., paternity confusion; Balme et al., 2013), this suggests that potentially infanticidal nonfathers were visiting family sites. An additional possibility is that male leopards which overlapped with family groups may have been related subadult males from previous litters; male philopatry has been observed in other high‐density leopard populations (Fattebert et al., 2015). Avoidance interactions could be weaker among families and recently dispersed offspring compared with older unrelated resident males.

### 4.2 Temporal activity patterns

Leopards at TNP displayed cathemeral daily activity patterns with peaks at dusk and dawn, similar to other leopard populations across a range of habitats including tropical forest and montane environments (Havmøller et al., 2020; Saisamorn et al., 2019; Van Cleave et al., 2018). Importantly, temporal activity patterns can vary significantly throughout the year in response to the variation in these environmental variables (Ota et al., 2019). Therefore, our findings represent only a snapshot of the summer diel activity patterns of this leopard population.

We also found intraspecific variation in daily activity patterns, with lone females and families allocating less activity to the nocturnal period compared to male leopards. This is consistent with the findings of Havmøller et al. (2020) who found female leopards in Tanzania were more diurnal compared to males. Higher daytime activity of females could arise from of avoidance of male leopards, or the tracking of prey with different activity patterns to the preferred prey of male leopards (Azevedo et al., 2018; Rostro‐García et al., 2018). The daily consumption rate of a young female leopard was nearly half that of adult males at TNP (2.4 vs. 4.3 kg; Farhadinia, Johnson, Hunter, et al., 2018); it is therefore plausible that female leopards in this area hunt smaller diurnal prey such as Afghan pika (*Ochotona rufescens*) and birds.

In terms of temporal overlap between leopard groups, both combinations of males/lone females and males/families exhibited high overlap (∆ = 0.80 and 0.79, respectively). However, there was only evidence for different activity curves between males and females, which can be explained in two ways. First, the smaller sample size of family detections (*n* = 34) compared to other groups can introduce considerable uncertainty to estimates of activity using kernel density estimation (Lashley et al., 2018). Therefore, caution should be exercised when interpreting activity curve overlap between leopard families and males due to the small number of detections.

Alternatively, it is possible that temporal avoidance was present, but the scale of our analysis was too coarse to detect avoidance patterns. The reliability of common methods for detecting temporal avoidance is determined by the strength of avoidance patterns, with weaker avoidance behaviors less likely to be detected (Niedballa et al., 2019). Avoidance responses operating at a smaller temporal scale (e.g., hours) may not be detected by our analysis. The leopard population at TNP exists at high densities where leopards are likely to encounter each other more frequently than compared to a lower density population, raising the possibility that any temporal avoidance could be operating at a finer scale. In this case, avoidance may operate at a shorter temporal scale as “reactive” behaviors (e.g., fleeing after a direct interaction), rather than long‐term temporal avoidance (Dröge et al., 2017). With a larger dataset with more detections, methods such as time‐to‐event analyses (Swanson et al., 2016) could be appropriate to investigate fine‐scale intraspecific interactions from camera‐trap data.

## 5 CONCLUSIONS

Although we did not find evidence that leopard families partition their space use and temporal activity in response to the presence of males, avoidance patterns may not have been detected due to the small sample size in our study. Therefore, to determine whether infanticide is an important driver of space use in this population, repeated camera trapping of families in this area during the summer months to maximize the number of detections of families would allow future studies to clarify the behavioral patterns of this understudied group. Accounting for intraspecific variation which may be overlooked by models which treat a species as a homogenous group can have important implications for conservation, as threats may not affect all individuals equally. Our study indicated that lone females and families are more active during daylight hours compared to males, potentially disproportionately exposing these groups to anthropogenic threats, which are more likely to occur during the day, such a poaching.

## CONFLICTS OF INTEREST

The authors have no conflicts of interest to declare.

## AUTHOR CONTRIBUTION

**Sarah Rouse:** Conceptualization (equal); Data curation (equal); Formal analysis (equal); Investigation (equal); Methodology (equal). **Pouyan Behnoud:** Data curation (equal). **Kave Hobeali:** Data curation (equal); Project administration (equal). **Peyman Moghadas:** Data curation (equal); Investigation (equal); Project administration (equal). **Zolfaghar Salahshour:** Data curation (equal). **Hossein Eslahi:** Data curation (equal); Project administration (equal). **Mousa Ommatmohammadi:** Data curation (equal); Project administration (equal). **Ali Khani:** Data curation (equal); Project administration (equal). **Abolfazl Shabani:** Project administration (equal). **David W. Macdonald:** Conceptualization (equal); Funding acquisition (equal); Methodology (equal); Project administration (equal); Resources (equal); Supervision (equal); Validation (equal); Writing‐review & editing (equal). **Mohammad S. Farhadinia:** Conceptualization (equal); Data curation (equal); Formal analysis (equal); Funding acquisition (equal); Investigation (equal); Methodology (equal); Project administration (equal); Validation (equal); Visualization (equal).

## Supporting information

Appendix S1

## Data Availability

Datasets analyzed during the current study are available on Figshare as (https://figshare.com/s/8f2846cb4bb2f52ef567).
