# Supplementary material for: Intraspecific interactions in a high‐density leopard population
Source: Ecol Evol. 2021 Nov 10;11(23):16572–84. doi: 10.1002/ece3.8227 (PMC8668769; doi:10.1002/ece3.8227)
Supplement: Supplementary file 1 — Appendix S1 [file ECE3-11-16572-s001.docx]

**Intraspecific interactions in a high density leopard population**

Sarah Rouse^1^, Pouyan Behnoud^2^, Kaveh Hobeali^2^, Peyman Moghadas^2^, Zolfaghar Salahshour^3^, Hossein Eslahi^3^, Mousa Ommatmohammadi^3^, Ali Khani^3^, Abolfazl Shabani^3^, David W. Macdonald^4^ and Mohammad S. Farhadinia^2 5^ *

^1^School of Geography and the Environment, University of Oxford, Oxford, UK

^2^ Future4Leopards Foundation, Tehran, Iran.

^3^ Khorasan Razavi Provincial Office of Department of the Environment, Mashhad, Iran.

^4^ Wildlife Conservation Research Unit, University of Oxford, Tubney House, Oxfordshire, OX13 5QL, Oxford, U.K.

^5^ Oxford Martin School and Department of Zoology, University of Oxford, 34 Broad Street, Oxford OX1 3BD, UK.

* Corresponding author email: [sarah.rouse97@googlemail.com](mailto:sarah.rouse97@googlemail.com)

**Table A1.** The four possible conditional occupancy states for a male/lone female occupancy model, where Ψ = probability of occurrence given a particular combination of presence/absence. Occupancy states for the male/family occupancy model were the same, with families in place of females.

| Occupancy state | Description | Male | Female |
| --- | --- | --- | --- |
| Ψ_11_ | Male and female present | 1 | 1 |
| Ψ_10_ | Male present, female absent | 1 | 0 |
| Ψ_01_ | Male absent, female present | 0 | 1 |
| Ψ_00_ | Male and female absent | 0 | 0 |

| Covariate | Description | Hypothesis |
| --- | --- | --- |
| Latitude  (Ψ) | Latitude (decimal degrees) of camera station | Geographic control (Rota et al., 2016; Miller et al., 2018) |
| Longitude (Ψ) | Longitude (decimal degrees) of camera station | Geographic control (Rota et al., 2016; Miller et al., 2018) |
| Prey index  (Ψ) | Index of prey abundance at each camera station | Leopard occupancy positively associated with prey availability for all leopard groups, highest for families which are more dependent on the resource (Marker & Dickman, 2004) |
| Distance to edge  (Ψ) | Distance (metres) from camera station to nearest boundary of Tandoureh National Park  (min = 0m, max = 7453m) | Family occupancy negatively associated with proximity to park edge to minimise interaction with human disturbance |
| Trail  (Ψ, p) | 0/1 variable indicating whether camera station was placed at a trail (0) or water source (1) | **Ψ** Expected no difference between males and lone females, but higher occupancy expected for leopard families which might anchor to resource-rich areas  **p** Detection probability higher at water stations compared to trails (Farhadinia et al. 2019) |
| DaysActive  (p) | Number of days (out of 56) that camera was active across the sampling period | Detection probability positively associated with number of days camera was active |

**Table A2.** Occupancy (Ψ) and detection (p) covariates included in the multispecies occupancy models with their associated *a priori* hypotheses.
